# Comparison of Different Final Impression Techniques for Management of Resorbed Mandibular Ridge: A Case Report

**DOI:** 10.1155/2014/253731

**Published:** 2014-08-10

**Authors:** Bhupender Yadav, Manisha Jayna, Harish Yadav, Shrey Suri, Shefali Phogat, Reshu Madan

**Affiliations:** Department of Prosthodontics, SGT Dental College and Hospital, Gurgaon 122001, India

## Abstract

The history of complete denture impression procedures has been influenced largely by the development of impression materials from which new techniques and ideas arose. The purpose of this study was to compare the retention of complete dentures made by using different impression techniques like conventional, admixed, all green, and functional techniques. The results showed that there was significant difference in retention between the six techniques where functional technique showed the highest mean value of retention followed by elastomeric, all green, and admixed, while cocktail and green stick compound showed the lowest mean value. However, on clinical examination, the retention produced by the six techniques was satisfactory.

## 1. Introduction

Complete dentures are primarily mechanical devices but since they function in the oral cavity, they must be fashioned so that they are in harmony with the normal neuromuscular function. The wearing of complete dentures may have adverse effects on the health of both oral and denture supporting tissues [1]. Residual ridge resorption is a complex biophysical process and a common occurrence following extraction of teeth. Ridge atrophy is most dramatic during the first year after tooth loss followed by a slower but more progressive rate of resorption thereafter [2, 3]. Making a definitive impression of an edentulous arch can be challenging when the residual ridges present are less than ideal.

Due to the anatomical differences between the maxilla and the mandible, as well as the differences in primary and secondary load-bearing areas, impressions of resorbed mandibular ridges require special considerations [4]. Mandibular residual ridges with adequate bone support can usually be precisely recorded with conventional impression techniques using materials such as zinc oxide eugenol (ZOE) or elastomeric impression materials because of the inherent accuracy of these materials and their propensity to distribute pressure equally [5]. As the residual ridges resorb, the tissues become unsupported and displaceable; the use of conventional impression techniques will result in a distorted impression. Therefore, the impression technique needs to be modified.

A number of modified impression techniques for resorbed mandibular ridge have been suggested by various authors such as admixed [6], functional [7], all green [8], and cocktail technique [9]. All these techniques capture the primary and secondary load-bearing areas without distortion of the residual ridge. The use of these impression techniques has the following advantages: (1) they can be easily controlled to gain maximum coverage; (2) they can be corrected readily; (3) they can be used to accurately determine the extent of the mucobuccal reflections; and (4) they can be used to direct pressure toward the load-bearing areas, specifically, the buccal shelf and the slopes of residual ridges in the mandible [10].

The web search for resorbed mandibular ridges on the PubMed results in listing of a majority of articles on modified impression techniques to fabricate complete dentures, but there was hardly any case report which compares all these different techniques. This paper presents a clinical report to compare the retention of mandibular complete denture in resorbed mandibular ridge made by using six different final impression techniques.

## 2. Materials and Method

**Figure 1:**
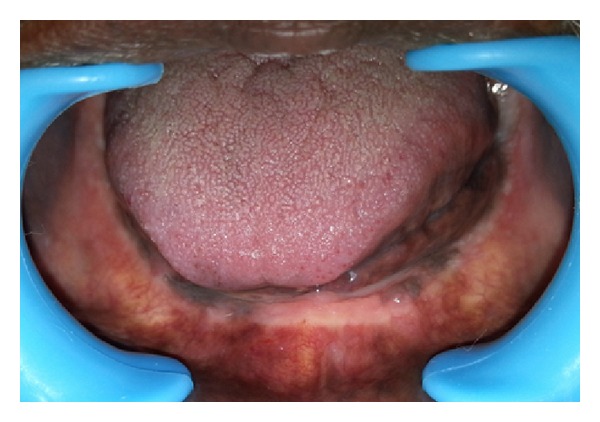
Resorbed mandibular ridge.

A 60-year-old male patient presented with the chief complaint of difficulty in mastication, loosening of upper and lower dentures, and poor esthetics for the past 4-5 years. He also complained of denture moving during swallowing and speaking. On intraoral examination, mandibular ridge was resorbed (Figure 1). There was no hyper mobile tissue on palpation.

The patient was informed of all the possible treatment modalities available; since he could not afford implant treatment, fabrication of complete denture was considered. The patient was apparently in good general health and did not report any systemic disease. The patient was informed about the study and an informed consent was taken.

Preliminary impression of the edentulous arch was made using irreversible hydrocolloid impression material (Vignette Chromatic, Dentsply, Gurgaon, India) in a metal stock tray. Impression was poured in type III dental stone (Stone Plaster, Neelkanth Minechem, Rajasthan, India). Custom trays were fabricated on the preliminary cast using self-cure acrylic resin (Rapid Repair, Dentsply, Gurgaon, India) tray material. Border extensions of the trays were adjusted to be at least 2 mm short of the vestibules on the preliminary cast. Maxillary border moulding was done using low fusing impression compound (green stick) (DPI Pinnacle Tracing Sticks, the Bombay Burmah Trading Corporation, Mumbai, India) and final impression was made using zinc oxide eugenol impression material (DPI Impression Paste, the Bombay Burmah Trading Corporation, Mumbai).

**Figure 2:**
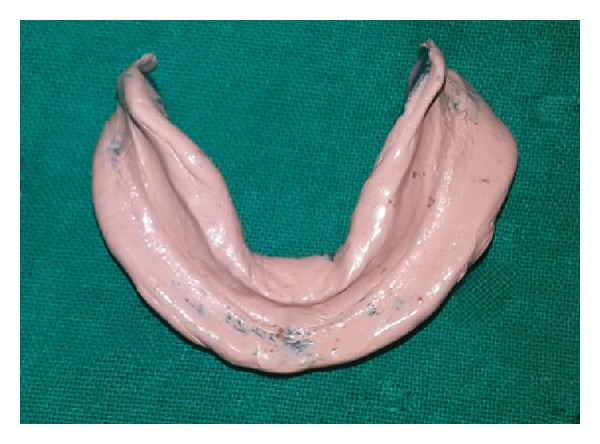
Conventional technique.

Mandibular border moulding and final impressions were made using six different techniques. In the first technique mandibular secondary impression was made using conventional method [11], that is, zinc oxide eugenol wash impression after border moulding with green stick compound in open mouth position (Figure 2).

**Figure 3:**
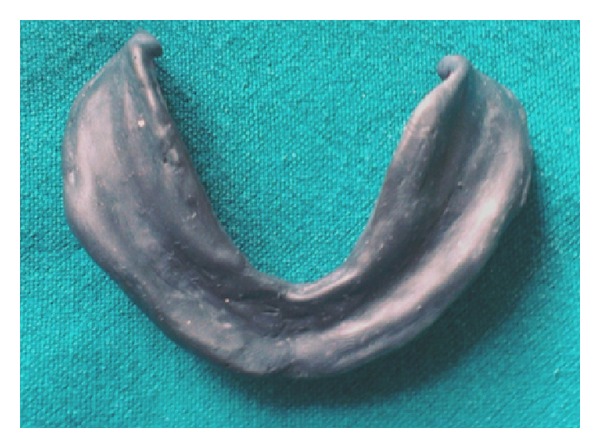
Admixed technique.

In the second technique, mandibular secondary impression was made using McCord and Tyson's admixed technique [6] for flat mandibular ridges. Impression compound (*DPI* Pinnacle, The Bombay Burmah Trading) and green tracing stick compound in the ratio of 3 : 7 parts by weight are placed in a bowl of water at 60°C and kneaded to a homogenous mass that provides a working time of about 90 seconds. Wax spacer is removed; this homogenous mass is loaded and patient is made to do various tongue movements (Figure 3).

**Figure 4:**
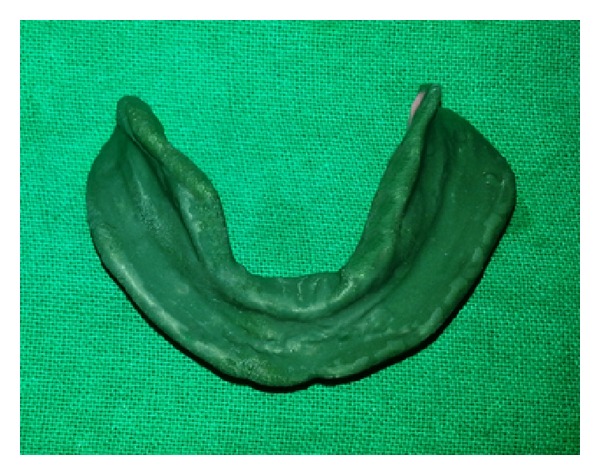
All green technique.

In the third technique, mandibular secondary impression was made using all green technique [8]. Green stick compound was kneaded to a homogenous mass and was loaded on the special tray and border movements were done (Figure 4). Final impression was made using zinc oxide eugenol.

**Figure 5:**
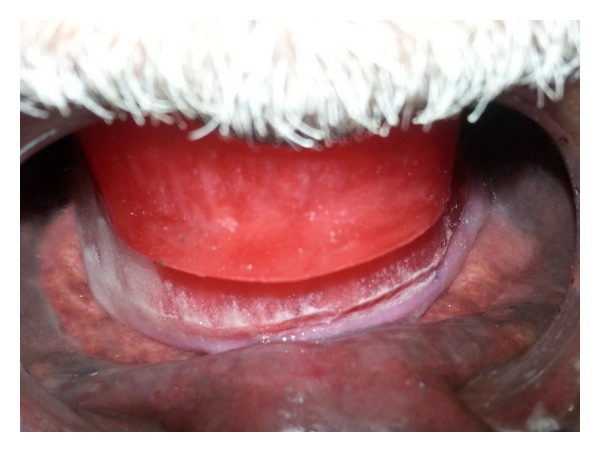
Functional technique.

Fourth technique was closed mouth functional impression technique by Winkler [7]. In this technique, denture bases with occlusal rim were fabricated on primary cast. Jaw relations were done to record appropriate horizontal and vertical dimensions. Tissue conditioning material was applied on the tissue surface of mandibular denture base and patient was asked to close the mouth in the prerecorded vertical dimension and do various functional movements such as puffing, blowing, whistling, and smiling (Figure 5). Three applications of tissue conditioner material were done at an interval of 8–10 minutes and functional movements were made by the patients. Final impression was made with light body addition silicone material with closed mouth technique.

**Figure 6:**
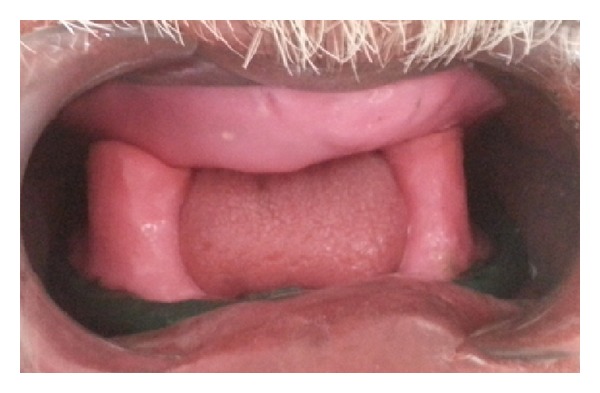
Cocktail technique.

Fifth technique was cocktail impression technique [9]. In this technique customized tray is fabricated with autopolymerizing acrylic resin according to Dynamic Impression Technique. A tray with 1 mm wax spacer and cylindrical mandibular rests in the posterior region is made at increased vertical height. Patient is advised to close his mouth so that the mandibular rests fit against the maxillary alveolar ridge. This helps to stabilise the tray in position by preventing anteroposterior and mediolateral displacement of the tray during definitive impression (Figure 6). Lingual surfaces of mandibular rests are made concave to provide space for the tongue to move freely during functional movements. McCord and Tyson's technique for flat mandibular ridges is followed for definitive impression. For recording the functional state, patient was instructed to run his tongue along his lips, suck in his cheeks, pull in his lips, and swallow by keeping his mouth closed, as in closed mouth impression technique, till the impression material hardens. The retrieved impression was visually inspected for surface irregularities and disinfected and was poured in dental stone.

**Figure 7:**
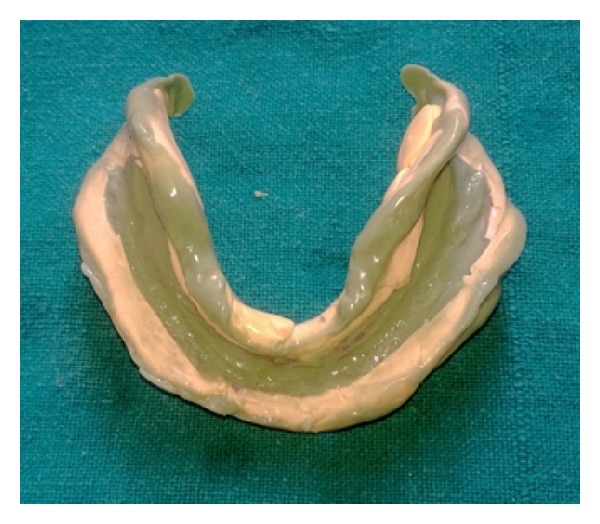
Elastomeric technique.

In the sixth technique, mandibular secondary impression was made using elastomeric impression materials. Tray adhesive (UPS Tray Adhesive, 3M ESPE, Seefeld, Germany) is applied over the border, internal and external surface of the acrylic custom tray, to facilitate the retention of the silicone border moulding material. An addition silicon putty material (Express TM Putty Soft, 3M ESPE, Seefeld, Germany) with an extended working time is loaded along the borders of special tray. The special tray is placed in the mouth and is border molded; the patient is asked to move the tongue according to standard impression procedures. The tray is removed from the mouth, and the impression is examined. Light-body addition of silicon impression material (Express TM light body, 3M ESPE, Seefeld, Germany) is loaded in the impression and inserted in the mouth. The patient is instructed to repeat the tongue movements, more vigorously, while the light-body impression material is border molded along the buccal and labial flange areas. After the material has set, the impression was removed from the mouth and examined for any discrepancy (Figure 7).

One maxillary master cast and 6 mandibular master casts were obtained after pouring the final impressions with type III dental stone (Stone Plaster, Neelkanth Minechem, Rajasthan, India). Maxillary master cast was duplicated to obtain five more maxillary master casts. Maxillary and mandibular occlusal rims were fabricated and jaw relations were recorded. Mounting was done on mean value articulator. Teeth arrangement was done and wax try-in was done in patient's mouth. Flasking, processing, finishing, and polishing of dentures are then done using conventional method [12]. Dentures were inserted in patient's mouth and were checked for retention, stability, support, and occlusion.

**Figure 8:**
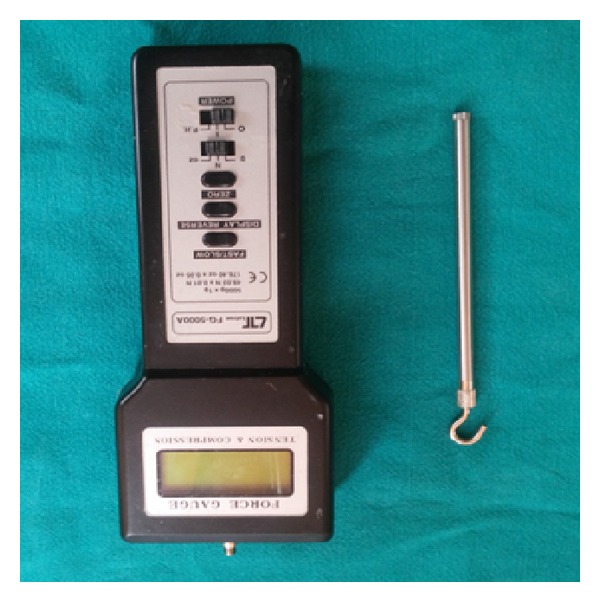
Digital force measurement gauge.

**Figure 9:**
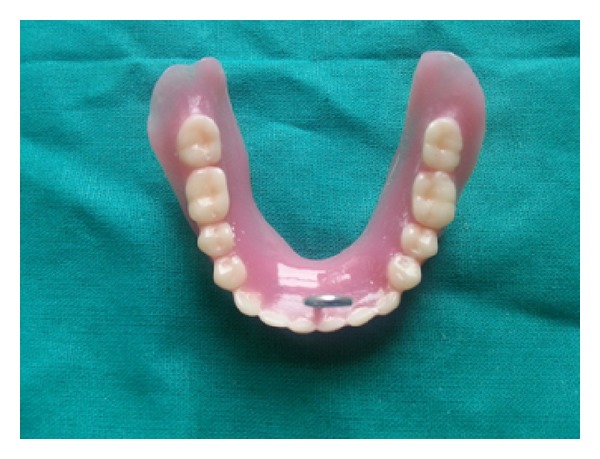
Denture with retention hook.

**Figure 10:**
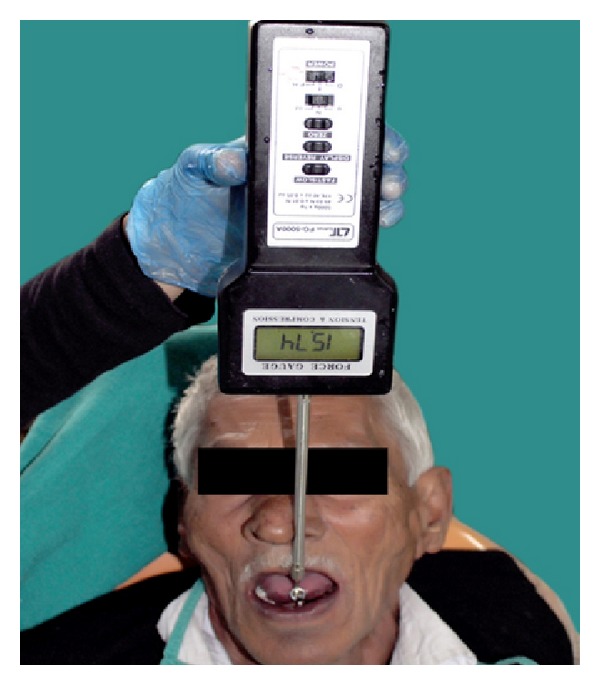
Patient with retention appliance.

To compare the retention between six mandibular dentures, the method used by Burns et al. [13, 14] using force measurement gauge (Figure 8) was used in this study. The patient was seated in the dental chair in an upright position with the head resting firmly against the headrest. The mandibular denture was positioned correctly on the tissues and the patient was asked to rest his tongue passively in the floor of the mouth with its tip adjacent to the anterior denture teeth. A wire loop (0.9 mm in diameter) was placed on the geometrical center of the polished lingual surface to which the pull end of the force meter (graduated up to 196 N) was attached (Figure 9). A vertical upward force was applied to dislodge the denture while the patient was sitting in an upright position with the occlusal plane parallel to the floor and the digital force measurement gauge (digital force gauge device model 47544, Extech Instruments Corporation) held in the palm of the hand (Figure 10). This force was measured in Newton and recorded as the denture's retention. Readings were recorded and the collected data was tabulated to evaluate and compare retention of the dentures fabricated using various techniques (conventional, admixed, putty rubber base and light body final wash, functional, cocktail, and all green). The retention of mandibular dentures was also evaluated clinically, and the patient was requested to comment on the retention of each mandibular denture.

## 3. Result

**Figure 11:**
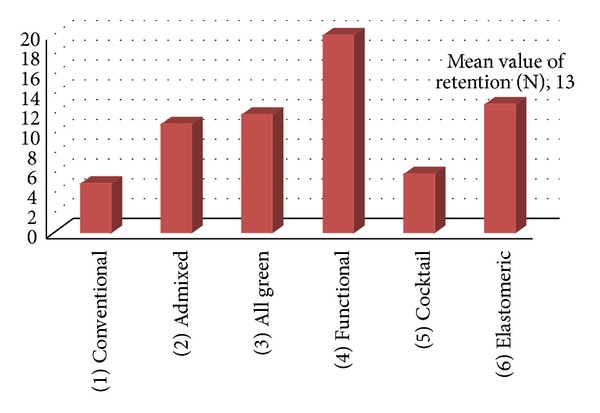
Mean value of retention for each final impression technique.

Three readings were recorded for each technique and a mean value was calculated. The mean force required to dislodge the mandibular dentures is shown in Figure 11. It was observed that the mean force required to dislodge the dentures was 5 N for green stick compound and metallic oxide wash, 11 N for admixed, 12 N for all green technique, 20 N for functional technique, 6 N for cocktail technique, and 13 N for putty and light body rubber base wash. The results showed that there was difference in retention between the six techniques where functional technique showed the highest mean value of retention followed by elastomeric, all green, and admixed while cocktail and green stick compound showed the lowest mean value. However, on clinical examination, retention of all the six mandibular dentures was found to be satisfactory and acceptable. Patient was most satisfied with the denture made from functional impression technique.

## 4. Discussion

The success of every complete denture relies on the fulfilment of the three basic properties of retention, stability, and support. Mandibular dentures usually present more difficulties in achieving these three properties, basically because of the larger number of anatomic limitations that requires added attention. The retention of the dentures is influenced by the factors like cohesion, adhesion, fluid, viscosity, atmospheric pressure, external factors arising out of oral-facial musculature, and occlusion (Murray & Darvell 1993; Shay 1997). The accuracy of complete denture impression techniques has been debated for many years. A wide diversity of denture border outlines, resulting from the use of the same impression procedure for all patients, has been shown and documented [15]. Numbers of impression techniques have been described in the literature for resorbed mandibular ridge [4, 16, 17]; each technique has its own advantages and disadvantages.

The degree of muscular activity and the region to which the denture can be extended without displacement are important aspects of any impression technique. For individuals with an accentuated bone resorption, it is difficult to obtain good retention and stability of the complete denture due to the presence of muscular insertions near the ridge crest or border, which might cause muscular-induced displacement of the denture. In these cases, functional technique is highly recommended and as per the results in the present study mandibular denture fabricated using functional technique [7] showed the highest mean value of retention. The results of the study are in accordance with the study conducted by Drago [17] which concluded that mandibular denture bases constructed from closed mouth technique were more retentive than the open mouth techniques. The closed mouth functionaltechnique by Winkler has certain advantages; since it is time saving, interference due to tray handling is eliminated; also there are less chances of under- or overextensions as movements are performed by the patient and pressure applied by the patient during impression making is the same as the pressure applied while occluding. However, there are certain disadvantages of this technique such as the fact that the dentist has no control over patient movement which may result in under- or overextended borders and also tongue is restricted to move anteriorly which may alter the anatomy of lingual border.

Elastomeric impression material has the advantage of single step border moulding using putty and accurately recording the minute details during final impression using light body. These properties were consistent with the results as the retention of the mandibular denture fabricated after final impression with elastomeric impression showed the second highest mean value of retention. In literature, various authors have recommended the use of elastomeric impression materials for border moulding and final impressions. Smith et al. [18] described a technique using a polyether impression material for border moulding the complete denture impression trays. The major advantages of this technique were that the border moulding could be accomplished in one-step and that the patient's functional movement was used in forming the borders. Tan et al. [19] concluded that polyether impression material requires less time to complete the border moulding process; the border recorded was longer and of less operator variability when compared with modelling plastic. Lu et al. [20] and Applebaum et al. [21] concluded that polyvinylsiloxane putty and light-body impression material are well suited for making complete denture impressions. Good results are obtained with less expenditure of time as well as less discomfort and inconvenience for the patient, especially in the hands of an inexperienced operator.

Cocktail technique [9] used eliminates the disadvantages of Winkler technique [7] as the customized tray that is fabricated in this technique has the advantage of avoidance of dislocating effect of the muscles on improperly extended denture borders and complete utilization of the possibilities of active and passive tissue fixation of the denture. Mandibular rests that fit against the maxillary alveolar ridge offer the advantage to stabilize the custom tray by preventing horizontal displacement of the tray during definitive impression. These features of the tray directly result in the impression material being shaped by the functional movements of the muscles and muscle attachments that border the denture base. Admixed technique [6], as recommended by McCord and Tyson for atrophic mandibular ridges used, has the advantage of recording the functional position of the muscles in a single step; also it requires less chair side time and is economical as compared to tissue conditioner or reline material.

Various surveys [22, 23] show that modelling plastic impression compound and zinc oxide eugenol impression paste are the most popular materials used for complete denture impression because of their fast setting, capability of reproducing fine details, easy handling and having no significant dimensional changes subsequent to hardening. However, although modelling plastic impression compound is an ideal impression material, it has certain limitations such as its short manipulation time and the fact that it hardens quickly in the mouth and does not remain in a plastic stage till the functional movements of the vestibular and alveololingual sulcular tissues are completed. Woelfel et al. [14] reported that it required an average of 17 placements to obtain a maxillary final impression using modelling plastic as the border moulding material. These limitations of the conventional method are consistent with results of the present study as the mandibular dentures fabricated using conventional method showed the least mean value of retention.

The variety of impression materials and the range of working characteristics of these materials made it possible to develop impression procedures best suited for specific conditions in each area in a given mouth. Whatever method is used for making impression, it should be based on the basic principles [24] of maximum tissue area coverage and intimate contact so as to achieve the objectives of retention, support, stability, esthetics, and preservation of ridge (supporting structures).

## 5. Conclusion

Prosthodontic rehabilitation of a patient with compromised residual ridge in a conventional manner is a difficult task. Modification in treatment procedure should be considered to fulfill the patient's functional and esthetic demands. This case report illustrates the impression techniques which are needed to achieve effective retention, stability, and support for Atwood's Order V and VI [24] ridge deformities. Mandibular denture made using functional impression technique showed the highest mean values of complete denture retention whereas denture made using green stick compound with metallic oxide final wash showed the lowest mean values of complete denture retention. There were certain limitations such as limited sample size (only 1 patient) and prolonged treatment time.
